# The effect of mobile application-based technology on post-stroke aphasia: a systematic review

**DOI:** 10.3389/fneur.2024.1405209

**Published:** 2024-06-12

**Authors:** Zihui Jiang, Mingping He, Chenchen Zhang, Xiuen Chen

**Affiliations:** ^1^Department of Rehabilitation Medicine, Huadong Hospital Affiliated to Fudan University, Shanghai, China; ^2^Department of Cardiology, Huadong Hospital Affiliated to Fudan University, Shanghai, China; ^3^Department of Rehabilitation Medicine, Shanghai Fourth Rehabilitation Hospital, Shanghai, China; ^4^School of Exercise and Health, Shanghai University of Sports, Shanghai, China; ^5^Department of Neurology, Yueyang Hospital of Integrated Traditional Chinese and Western Medicine, Shanghai University of Traditional Chinese Medicine, Shanghai, China

**Keywords:** mobile application, aphasia, speech and language therapy, post-stroke, systematic review

## Abstract

**Background:**

Enhancing speech-language therapy remains the most effective strategy for improving post-stroke aphasia, However, conventional face-to-face interventions often lack the necessary therapeutic intensity. In recent years, mobile application-based speech-language therapy has emerged progressively, offering new opportunities for independent rehabilitation among aphasic patients. This review aims to evaluate the impact of mobile application-based interventions on post-stroke aphasic.

**Methods:**

By conducting a systematic search across five databases (PubMed, Web of Science, EMBASE, CINAHL, and Scopus), we identified and included studies that investigated the utilization of mobile application-based technologies (such as computers, iPads, etc.) for treating post-stroke aphasia.

**Results:**

This study included 15 research investigations, including 10 randomized controlled trials (RCTs), four self-controlled studies and one cross-over experimental design study. Among these, eight studies demonstrated the efficacy of mobile application-based therapy in enhancing overall language functionality for post-stroke aphasia patients, three studies highlighted its potential for improving communication skills, three studies observed its positive impact on spontaneous speech expression. Moreover, four studies indicated its effectiveness in enhancing naming abilities, two studies underscored the positive influence of mobile application-based interventions on the quality of life for individuals with aphasia. Six studies noted that speech improvement effects were maintained during the follow-up period.

**Conclusion:**

The results of this review demonstrate the potential of mobile application-based interventions for improving speech-language function in individuals with aphasia. However, further high-quality research is needed to establish their effects across different domains and to delve into the comparative advantages of various treatment approaches.

**Systematic review registration:**

https://www.crd.york.ac.uk/prospero/display_record.php?RecordID=405248

## 1 Introduction

Aphasia arising post-stroke is an acquired communication disorder characterized by impairment in linguistic abilities. It stems from varying degrees of damage to the language center of the brain (usually located in the left hemisphere), which affects oral expression, reading ability, writing ability, language comprehension, and even cognitive and computational functions (1, 2). After an initial ischemic stroke, ~30% of patients may manifest symptoms of aphasia (3). Studies reveal that during the year following a stroke, ~43% of individuals with aphasia still confront ongoing communication challenges (4). Aphasia significantly hinders daily life functioning, subsequently reducing quality of life and potentially leading to issues such as depression (5–8).

In recent years, an increasing number of studies have focused on the use of mobile applications, such as computers and tablets, to enhance the expressive language skills of individuals with aphasia (9, 10). For example, Zhou et al. (11) found that a 14-day, 30-min-a-day computer-based training session resulted in more significant speech improvements compared to traditional treatments. Mobile application-based therapy is gaining attention as a means of remote delivery (12). One potential advantage of this treatment modality is its potential for increased cost-effectiveness, reduced therapist burden, and enhanced patient satisfaction and treatment adherence (13).

Three systematic review studies have already explored the impacts of various innovative technologies on aphasia, and their consistent findings suggest that innovative technologies hold promise in improving language functions among individuals with aphasia (14–16). However, the systematic reviews conducted by Lavoie et al. (14) and Russo et al. (16) focused only on the impact of a specific technology on language performance in a particular domain for people with aphasia and the literature included was limited to studies conducted prior to 2017. Additionally, the review by Repetto et al. covered literature from only three databases (15). Hence, the objective of this present systematic review is to comprehensively explore the impact of mobile application-based speech-language therapy (SLT) on language functional performance across multiple domains in individuals with aphasia.

## 2 Methods

### 2.1 Search strategy

This systematic review strictly follows the guidelines outlined by the PRISMA framework. The systematic review has been registered in the PROSPERO-International Prospective Register of Systematic Reviews (CRD42023405248). The search timeframe spans from the inception of databases to August 15, 2023. We conducted searches in five major databases (PubMed, Web of Science, EMBASE, CINAHL, and Scopus). To ensure search accuracy, we employed PubMed MeSH terms including “stroke,” “aphasia,” and “computer” to identify relevant keywords for retrieval. In the search process, Boolean operators were utilized to combine these keywords, aiming to capture a comprehensive range of literature (see Supplementary Table S1 for the search formula).

### 2.2 Study selection

Inclusion criteria were determined according to the PICOS principles (include participant, intervention, control group, outcomes and study): (1) Study design: English language, randomized controlled trial, self-controlled trial, and crossover design trial; (2) Study participant: individuals ≥18 years of age with stroke, confirmed by medical imaging diagnosis, and diagnosed by speech-language pathologists according to the diagnostic criteria of the Aphasia Scale (e.g., the Western Aphasia Battery of Tests); (3) Intervention method: mobile application technology, including, but not limited to, application interventions on devices such as PCs, iPads, tablets, and cell phones. If mobile application technology is used in combination with other treatments, the control group needs to adopt the same method. (4) Control group: the control group included no intervention (waiting group), therapist intervention alone, or computer-based pseudo-intervention; (5) The outcome measures include language functioning outcomes such as overall language function (assessed by the Overall Language Scale), functional communication skills (assessed by the Functional Communication Scale), spontaneous language functioning (assessed by picture description tasks, etc.), and naming ability (assessed by the Naming Scale). In addition, attention will also be paid to outcomes such as quality of life related to aphasia.

### 2.3 Literature screening procedures

Literature screened in the database will be imported into EndNote software. Two researchers will screen the titles and abstracts of the literature based on predetermined inclusion criteria. During the screening process, if disagreement arises between the two researchers, a third researcher will be asked to participate in order to jointly decide whether to include or exclude the literature. After the initial screening, the full-text screening stage was carried out. During the full-text screening process, evaluations are also made based on pre-set criteria. After the screening is complete, a reference search will be performed for the included literature to manually search for relevant literature that may meet the requirements.

### 2.4 Data extraction

The researchers will create data extraction tables to record the information from each included study, which will be populated into Tables 1, 2. Extracted details will include author, publication year, study type, sample size, type of aphasia, age, gender, duration of condition, intervention setting, severity of aphasia, intervention method, frequency and duration of intervention, description of control group, outcome measures, and results. Two researchers will carry out the data extraction process, and in case of any discrepancies, the opinion of a third researcher will be sought. Given the significant variations in intervention content, outcome assessment, and study designs across different studies, conducting a meta-analysis would not be appropriate.

**Table 1:** Characteristics of randomized controlled trials.

**References**	**Sample size**	**Age (year)**	**Sex (female) (%)**	**Post-stroke duration (d/m/y)**	**Type of aphasia**	**Severity of aphasia (mean ± SD)**	**Interventionaddress**	**Intervention**	**Session,duration**	**Control**	**Follow up**	**Outcomemeasures**	**PEDro score**
Braley et al. (17)	32 IG: 17 CG: 15	IG 58.9 ± 10 CG 62.4 ± 9.9	43.75%	IG 53 ± 56 (m) CG 38.1 ± 32 (m)	Broca's:10 Anomic:10 Conduction:6 Wernicke's:4 Transcortical Motor:2	WAB-AQ IG: 61.62 ± 24.28 CG: 66.02 ± 19.08	Home	iPad-based therapy	at least 30min/day,5 days a week for 10 weeks	Paper workbooks	2 weeks	WAB-R-AQ; WAB-R-LQ; WAB-R-CQ; SAQOL-39.	7
Cherney (18)	25 IG: 11 CG: 14	IG 56.6 ± 9.2 CG 61.1 ± 14.8	36%	IG 66.7 ± 71.5 (m) CG 41.3 ± 45.7 (m)	NA	WAB-AQ IG: 62 ± 19.9 CG:47.3 ± 27.9	Not clear	Computer-based therapy	1 h/session, 2–3 times a week, 24 times in total	Waitlist	NA	WAB; Discourse words/min; Discourse CIUs/min.	6
Elhakeem et al. (19)	50 IG: 25 CG: 25	IG 57.04 ± 10.88 CG 58.80 ± 11.58	20%	NA	Broca's:24% Anomic:2% Transcortical motor: 18% Transcortical mixed: 24% Global: 32%	BADE 0.8 ± 0.58	Clinic	Computer-based therapy	60 min/session,48 sessions over 6 months	Traditional speech and language therapy	NA	BADE	8
Kesav et al. (20)	20 IG: 11 CG: 9	IG 56.27 ± 11.62 CG 48.67 ± 11.83	30%	IG 31.2 ± 31 (d) CG 29.3 ± 30 (d)	Broca(50%) Wernicke(25%) Anomic(15%) Transcortical sensory aphasia(10%)	WAB-AQ IG: 32.4 ± 25.8 CG:45.1 ± 28.4	NA	Computer-based therapy combined with traditional therapy	120 min/session, 3 session a week for 4 weeks	Traditional speech and language therapy	8 weeks	WAB	6
Palmer et al. (21)	33 IG: 16 CG: 17	IG 69.5 ± 12.2 CG 66.2 ± 12.3	36.4%	IG 6.2 (y) CG 6.6 (y)	Fluent: 6 Non fluent: 25 Global: 2	Mild: 20 Moderate: 9 Severe: 4	Home	Computer word finding training	at least 20 min 3 days a week for 5 months	Usual care	3month	The change in word retrieval ability	5
Palmer et al. (22)	169 IG: 83 CG: 71	IG 64.9 ± 13.0 CG 63.8 ± 13.1	39%	IG 2.9 ± 2.9 (m) CG 3.6 ± 4.8 (m)	Non-fluent: 61 Fluent: 10 Mixed non-fluent: 31 Anomic: 52	Severity of word finding difficulty Mild: 44% Moderate: 30% Severe: 26%	Home	Computer word finding training combined with usual care	20–30min/session, 7 sessions a week for 6 month	Paper-based puzzle book activities and usual care	6month	Picture naming test of 100 personally relevant words; functional communication ability; COAST	8
Spaccavento et al. (23)	22 IG: 13 CG: 9	IG 57.38 ± 9.23 CG 64.11 ± 15.04	27%	IG 25.92 ± 25.99 (d) CG 20 ± 10.66 (d)	Global: 9 Broca: 8 Wernicke's: 1 Transcortical sensory: 2 Anomic: 2	Severe Aphasia: 12 Moderate Aphasia: 10	Clinic	Computer-based therapy	50min/session, 5 days a week for 8 weeks	Traditional therapist-mediated treatment	NA	AAT: FOQ-A: QLQA	6
Doesborgh et al. (24)	18 IG: 8 CG: 10	IG 62 ± 9 CG 65 ± 12	50%	>11 (m)	NA	NA	Clinic	Computer-based therapy	30–40min a session, 2–3 sessions a week for 8 weeks	No treatment	NA	BNT; ANELT-A	6
Katz and Wertz (25)	40 IG: 21 CG: 19	IG 61.6 ± 10 CG 64.4 ± 6	20%	IG 6.2 ± 5.2 (y) CG 5.4 ± 4.6 (y)	NA	WAB-AQ IG:68.9 ± 24.3 CG:72.2 ± 24.8	clinic	Reading Treatment Software	3 hours a week, for 26 weeks	Computer stimulation	NA	PICA; WAB-AQ	4
Cherney et al. (26)	32 IG: 19 CG: 13	IG 58.27 ± 18.55 CG 55.19 ± 11.46	40.6%	IG 39.75 ± 40.76 (m) CG 60.97 ± 30.19 (m)	Fluent: 18 Non-fluent:14	WAB-AQ IG: 59.21 ± 18.07 CG: 62.76 ± 16.81	Home	Computer-based treatment	90 minutes a day, six days a week for six weeks	Computer game, Bejeweled 2©	6 weeks	WAB-LQ	5

IG Intervention group; CG Control group; m, months; d, days; y, years; WAB, Western Aphasia Battery; R, Revised; AQ, Aphasia Quotient; CQ, Cortical Quotients; LQ, Language Quotients; SAQOL, Stroke and Aphasia Quality of Life Scale; CIU, correct information units; BADE, Boston Diagnostic Aphasia Examination; COAST, Communication Outcomes After Stroke questionnaire; AAT, Aachener Aphasia Test; FOQ-A, Italian Version of Functional Outcome Questionnaire for Aphasia; QLQA, Quality of Life Questionnaire for Aphasics; BNT, Boston Naming Test; ANELT-A, Amsterdam Nijmegen Everyday Language Test; PICA, The Porch Index of Communicative Ability; CETI, Communicative Effectiveness Index; ASHA-FACS, Functional Assessment of Communication Skills for Adults; CAT, Comprehensive Aphasia Test; CTPD, Cookie Theft Picture Description.

**Table 2:** Characteristics of quasi-experimental studies.

**References**	**Study design**	**Sample size**	**Age (year)**	**Sex (female) (%)**	**Post-stroke duration (d/m/y)**	**Type of aphasia**	**Severity of aphasia**	**Interventionaddress**	**Intervention**	**Session,duration**	**control**	**Follow up**	**Outcomemeasures**
Archibald et al. (27)	Self-controlled study	8	71 ± 11.1	25%	48.3 ± 53.27 (m)	Broca's:2 Anomic:3 Conduction:2 Global:1	WAB-AQ 60.29 ± 33.37	Home/clinic	Computer-based therapy	At least 1 hour/week, 15 weeks	NA	NA	WAB; CETI; ASHA-FACS
Choi et al. (28)	Self-controlled study	8	50.75 ± 8.3	50%	29.8 ± 25 (m)	Broca's:2 Wernicke's:3 mixed transcortical:1 anomic:1 Global:1	K-WAB-AQ 49.6 ± 26.38	Home	Ipad-based therapy	The number of treatments is not clear, 4 weeks	NA	One month	K-WAB-AQ
Zettin et al. (29)	Self-controlled study	7	46 ± 7.7	57%	49.7 ± 35.7 (m)	Non fluent: 7	WAB-AQ 42.1 ± 16.1	Home/clinic	Computer-based therapy	90 min per day, 5days a week for 6weeks.	NA	NA	WAB; BNT; picture description task
Kurland et al. (30)	Self-controlled study	21	66 ± 8.4	38%	29.3 ± 37.1 (m)	NA	Mild:8 Moderate:9 Severe:4	Home	Tablet-Based Home Practice	At least 20 min, 5–6 days per week, for 6 months	NA	4 months	Percent accuracy on naming.
Stark and Warburton (31)	Cross-over study	7	63.6 ± 13.88	37.5%	36.2 ± 25 (m)	NA	NA	Home	Self-delivered iPad speech therapy	20 min a session, 7 days per week, for 4 weeks	Computer game, Bejeweled©	6 month	CAT; content unit production and rate of speech on the CTPD.

IG, Intervention group; CG, Control group; m, months; d, days; y, years; WAB, Western Aphasia Battery; R, Revised; AQ, Aphasia Quotient; CQ, Cortical Quotients; LQ, Language Quotients; SAQOL, Stroke and Aphasia Quality of Life Scale; CIU, correct information units; BADE, Boston Diagnostic Aphasia Examination; COAST, Communication Outcomes After Stroke questionnaire; AAT, Aachener Aphasia Test; FOQ-A, Italian Version of Functional Outcome Questionnaire for Aphasia; QLQA, Quality of Life Questionnaire for Aphasics; BNT, Boston Naming Test; ANELT-A, Amsterdam Nijmegen Everyday Language Test; PICA, The Porch Index of Communicative Ability; CETI, Communicative Effectiveness Index; ASHA-FACS, Functional Assessment of Communication Skills for Adults; CAT, Comprehensive Aphasia Test; CTPD, Cookie Theft Picture Description.

### 2.5 Quality assessment

The Cochrane bias risk tool will be employed to analyze the risk of bias in the included randomized controlled trials (See content 1 in the Additional file for detailed judging details) (32). Evaluation of Six Bias Domains: Selection bias, Performance bias, Detection bias, Attrition bias, Reporting bias, and Other potential sources of bias. If <1 domain is assessed as high risk (-), the study is considered as low risk. If one or two domains are assessed as high risk (-) or unclear (?), the study is considered as medium risk. If more than two domains are assessed as high risk (-) or unclear (?), the study is considered as high risk (33). The quality of the included literature strictly followed the PEDro scale, which consists of 11 entries out of 10 points. Studies with a total score of ≥7 points will be classified as high quality, those with scores between 5 and 6 points as moderate quality, and those with scores ≤ 4 points as low quality. Assessment of Quasi-Experimental Studies Using the TREND (Transparent Reporting of Evaluations with Non-randomized Designs) Checklist (34, 35). This checklist comprises 22 items assessing the quality of titles and abstracts, introduction, methods, results, and discussion sections across five domains. The assessment will be carried out by two reviewers, and if consensus cannot be reached between them, a third reviewer will make the final decision.

## 3 Results

### 3.1 Study selection

**Figure 1:**
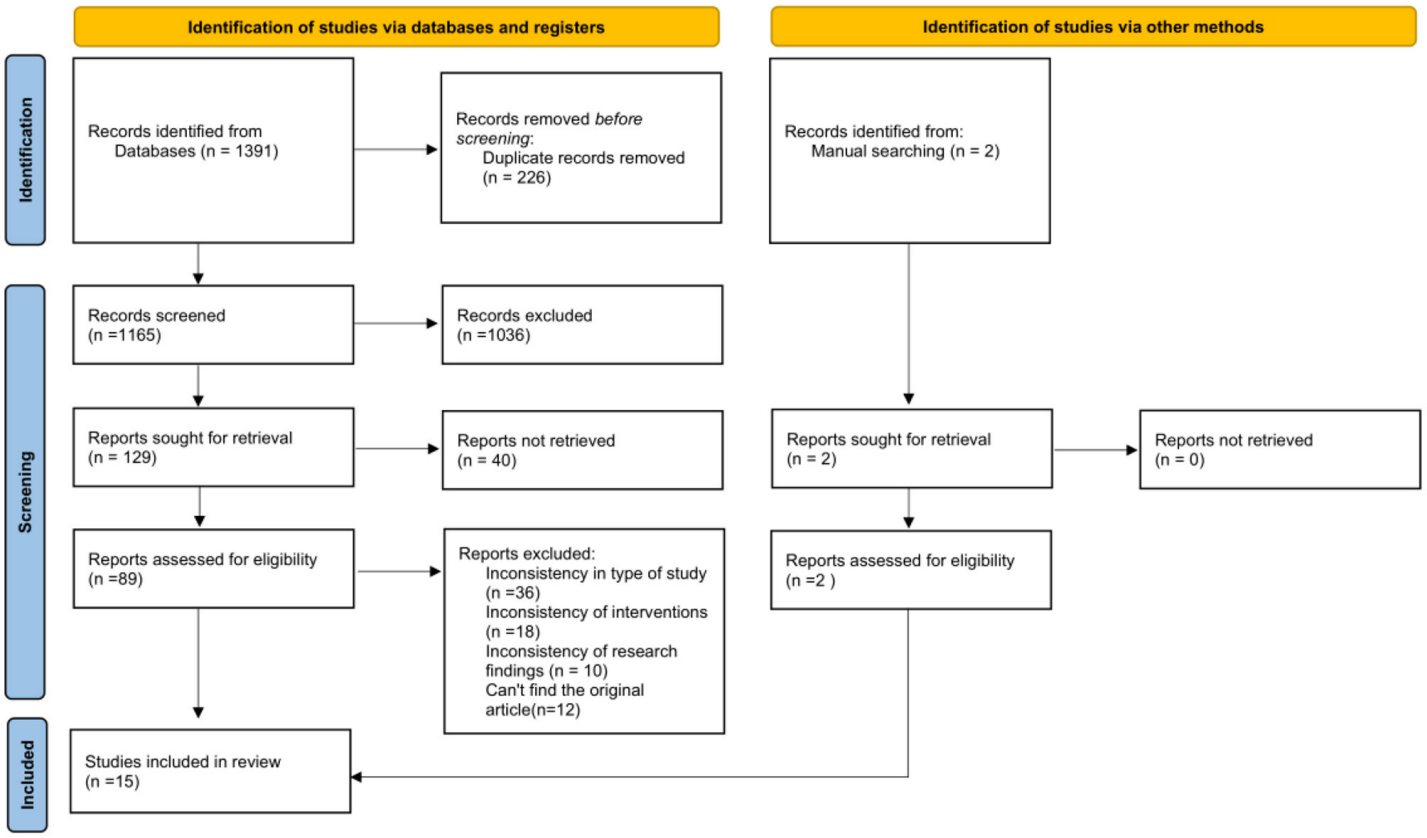
PRISMA flow chart.

After the initial search, a total of 1,391 documents were obtained. Through manual searching of the reference lists of the included literature, two additional papers were additionally identified. Subsequently, a full-text review of this literature was conducted, and a total of 89 studies met the review criteria. Ultimately, 10 randomized controlled trials, four self-controlled trail and one cross-over trial design study met the inclusion criteria for this study. The flow chart for literature screening is shown in Figure 1.

### 3.2 Quality of the studies

**Figure 2:**
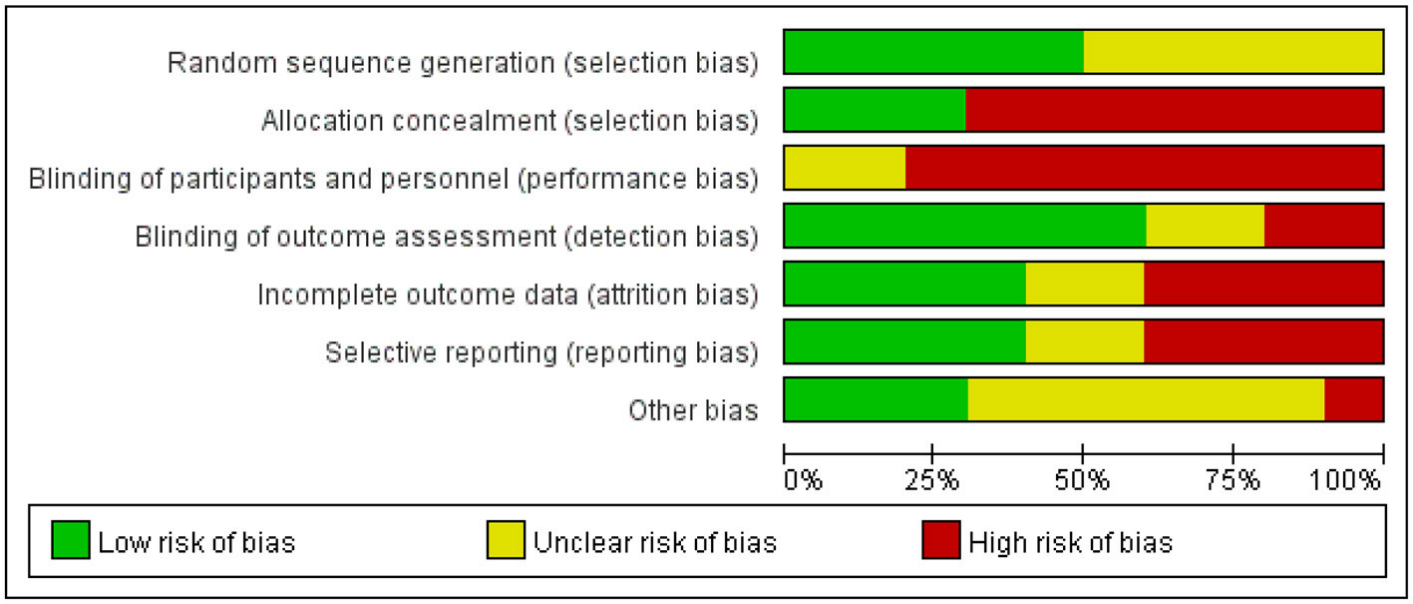
Risk of bias graph.

**Figure 3:**
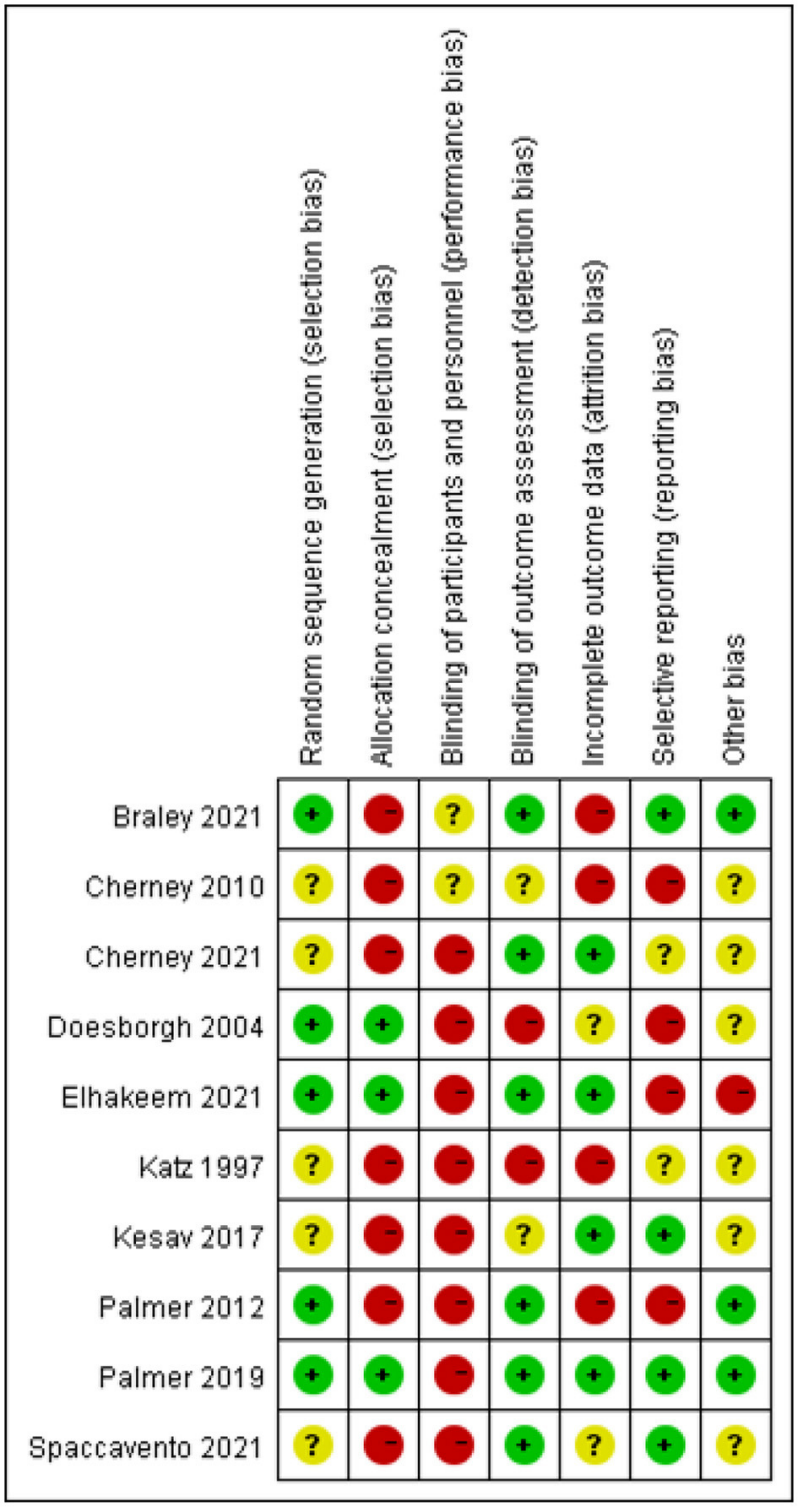
Risk of bias summary. + indicates low risk, - indicates high risk, ? indicates unclear risk of bias.

Ten randomized controlled trial studies were assessed for risk of bias and evaluated qualitatively. The results of the risk of bias analysis indicated that five studies performed random sequence generation, three studies described a hidden random allocation scheme, no studies blinded subjects and trial personnel due to the specificity of the trial intervention, six studies reported blinding the outcome assessor, and four studies demonstrated a low risk of attrition bias. Comparing the methodology of each study, only four studies had low reporting bias. Figures 2 and 3 depict total risk of bias plots for all randomized controlled trials, with one study at low risk and the remaining nine studies judged to be at high risk. By qualitative evaluation, three studies were of high quality, six studies were of moderate quality, and one study was of poor quality (details see Supplementary Table S2). Determining the quality of quasi-experimental studies is challenging, as evidenced by significant score disparities obtained using the TREND checklist. Evaluation using the TREND checklist revealed that included quasi-experimental studies performed well in aspects such as Title and Abstract (all studies met criteria), Background (all met criteria), Participants (eligibility criteria for participants), Intervention, Unit of Assignment, and Unit of Analysis. However, they showed poor performance in reporting results (patient registration and screening reports, adverse events, data analysis, follow-up, etc.) and discussion (see Supplementary Table S3).

### 3.3 Study characteristics

The characteristics of the 10 included randomized controlled studies are listed in Table 1. The sample size ranged from 18 to 169 individuals and covered a total of 441 subjects. The experimental group containing 224 subjects and the control group containing 217 subjects. In addition, 34.27% of the subjects were female, while the mean duration of stroke varied between 25 days and 6 years. The daily duration of the intervention varied between 20 min and 2 h, the intervention period varied from 4 weeks to 6 months, and the follow-up period was from 2 weeks to 6 months. Table 2 gives the characteristics of the four self-controlled studies and one cross-over experimental design study.

### 3.4 Characteristics of intervention component

#### 3.4.1 Single-component interventions

Eight studies used a single component of program intervention, focusing on 1–2 aspects of language. Of these, 3 studies used a method in which a person with aphasia watched an in-computer speech therapist read sentences and follow along (18, 26, 29). Four studies used interventions that targeted naming functions (21, 22, 24, 30). For example, Palmer et al. (22) designed a computer program containing 100 words related to the subject and had participants perform word-finding training. One other study focused on intervention methods in reading (25).

#### 3.4.2 Multi-component interventions

Seven studies used therapeutic procedures oriented toward multiple aspects of language (17, 19, 20, 23, 27, 28, 31). These treatments include auditory comprehension, reading comprehension, repetition, naming, and writing et al. These treatments address various aspects of language comprehension, naming, repetition, and spontaneous language.

### 3.5 General overview of technology

These studies primarily employed two types of technology: computers (in a total of 11 studies) and tablet devices (in a total of four studies). Compared to traditional face-to-face therapist-led interventions, computer and tablet-based interventions offer the advantage of structured difficulty progression and the ability to adjust difficulty levels based on participants' training performance. In this regard, 8 studies provided detailed descriptions of the difficulty levels within their treatment programs. Furthermore, computer- and tablet-based therapy programs typically provide only a single type of feedback. In this regard, only six studies have reported how computers provide feedback on the training performance of patients with aphasia, usually by providing feedback on how well the patient answered questions correctly. A detailed description of the content of the intervention can be found in the Supplementary Table S5.

### 3.6 Effects of interventions

#### 3.6.1 Overall language function

A total of 10 studies assessed the effect of intervention components on overall speech function improvement. Measurement tools used included the WAB, the AAT, and the BADE. Detailed results of the included literature (see Supplementary Table S4).

Eight of these 10 studies found that mobile application-based intervention content was effective in improving the overall speech performance of individuals with aphasia. The intervention methods used in these studies varied, with four of the studies utilizing a multi-component intervention approach. Additionally, four studies used a single-component intervention. Among these four studies, three studies utilized computer-based therapy with the imitation of a language therapist for reading tasks (18, 26, 29). One study used a reading function-specific approach (25). Of these eight studies, two found that the mobile application-based treatment was superior to the control group, with improvements in post-intervention language performance showing significant differences between groups. Katz and Wertz (25) reported a significant increase in WAB-AQ scores (*p* < 0.008) for computer-based reading software training compared to an active control group (computer stimulation). Braley et al. (17) found a significant improvement in WAB-R-AQ scores (*p* < 0.05) for 10 weeks of iPad-based SLT compared to home practice booklet training. Among these eight studies, four studies observed significant differences in overall language performance within the experimental group before and after treatment. However, no significant differences were observed when comparing between the experimental and control groups.

Of these 10 studies, two found that the intervention component of the experimental group failed to significantly improve language performance in aphasic patients. In one of these studies, although a significant difference between groups was found, the control group (using a traditional therapist intervention) showed a more significant improvement.

#### 3.6.2 Functional communication skills

Five studies assessed the impact of mobile application-based intervention methods on functional communication skills. The assessment instruments used were highly variable and included The Functional Assessment of Communication Skills for Adults, functional communication ability (using the activity scale of the Therapy Outcome Measures), Functional Outcome Questionnaire for Aphasia (FOQ-A), The Porch Index of Communicative Ability(PICA), Amsterdam Nijmegen Everyday Language Test (ANELT).

Of the five studies, two noted that functional communication skills improved after the intervention but were not significantly different from the control group, one used a blank control and the intervention was a single-content intervention (naming training), and one used a negative control and the intervention used was a multi-component intervention.

One study found that the experimental group's communication skills improved significantly after the intervention, with a significant difference between the groups (25). The two studies found no significant improvement in the experimental group's communication skills after the intervention, which used a multi-component intervention approach and a single-component intervention (word find).

#### 3.6.3 Spontaneous language

Three studies evaluated the impact of a mobile application-based intervention approach on spontaneous language production ability. In terms of evaluation, all of these studies used methods that described pictures and counted the number and frequency of words produced. Two of the studies used a non-randomized controlled study design and showed a significant increase in the number and frequency of words produced by people with aphasia after the intervention (29, 31). One other study used a randomized controlled trial design and found an increase in the number and frequency of words produced in the computer-based intervention group compared to the wait-for-treatment group, but there was no significant difference between the two groups (18).

#### 3.6.4 Naming ability

Five studies evaluated the effects of mobile application-based interventions on naming ability. Four studies found significant improvements in naming ability in individuals with aphasia following mobile application-based interventions. Two of these studies found significant differences in naming ability between groups using a single-component intervention method (word finding training). Two self-controlled studies found significant improvements in naming ability after the intervention. One RCT found no significant differences in naming ability between the experimental group and a blank control group after computer-based cued naming training.

#### 3.6.5 Quality of life

Three studies assessed the impact of mobile application-based interventions on quality of life. Two studies noted significant improvements in quality of life after the intervention, but there were no significant differences between groups compared to the control group, and one study found no significant improvement in quality of life after the intervention.

#### 3.6.6 Maintaining the effect

Eight studies explored the effects of mobile application-based interventions on the maintenance of efficacy, with two self-controlled studies and one crossover pilot study reporting the ability to maintain improvements in WAB-AQ (p=0.206), naming ability, and expressive ability in spontaneous speech in patients with aphasia after the time of follow-up (1, 4, and 6 months, respectively) (28, 30, 31). Three randomized controlled studies have found that WAB, naming ability in patients with aphasia was found to maintain its improvement at 6 weeks, 3 months, and 6 months of follow-up (21, 22, 26).

## 4 Discussions

Interventions based on mobile applications have gained increasing popularity for individuals with aphasia, primarily utilizing computers and tablets. These electronic devices have become integral to people's lives and have also brought convenience to rehabilitation therapy. Among the studies included, 66% allowed aphasic patients to use software for training at home, while therapist involvement during clinic-based training was primarily focused on addressing technical issues rather than guiding the therapy process. This underscores the potential for individuals with aphasia to engage in speech-language therapy either independently or with minimal assistance. A systematic review of 10 randomized controlled trials, four pre-post controlled studies, and one crossover trial indicates a high risk of bias. Heterogeneity of applications, varied outcome measures, differing intervention intensities, variations in aphasia onset times and severity, result in diverse study outcomes. This makes it challenging to assess and conduct meta-analyses across studies (36). Most of the studies had selection bias, all randomized controlled trials used random allocation, while five of them did not specify the exact method of randomization (18, 20, 23, 25, 26), leading to uncertain risks, and 70% of studies did not conceal allocation. There were no studies describing blinding of subjects, thus introducing performance bias. Monitoring bias had a better performance with 8 studies reporting assessor blinding, but 2 did not describe blinding procedures (18, 25). The five non-randomized controlled studies showed significant bias, lacking random allocation (27–31). Furthermore, according to the TREND report, none of the 5 studies described blinding. Thus, overall high bias across all studies is unfavorable for determining the effectiveness of mobile application-based speech-language therapy. However, overall, mobile application-based SLT holds significant promise and potential for improving the performance of individuals with aphasia.

The results of the study revealed that eight studies indicated the effectiveness of mobile application-based speech-language therapy (SLT) in improving the overall language functioning of individuals with aphasia. Of these, four studies showed no significant difference in improvement between the intervention and control groups. Mobile application-based therapy shows promise in enhancing language function; however, it is uncertain whether it is superior to the effects of traditional therapy. There were inconsistent results from five studies regarding whether mobile application-based therapy could produce transferable effects in terms of communicative and expressive language skills.

Four of the 10 RCTs compared mobile application-based speech-language therapy with Speech therapist (ST)-mediated therapy (19–21, 23). These studies aimed to explore whether computer-based interventions were comparable or potentially superior to face-to-face ST interventions. Among these, 3 studies found that computer-based interventions had outcomes for aphasic patients that were either better than or non-inferior to those of ST-mediated treatment. However, a study by Kesav et al. (20) reported that a regimen of three sessions per week for 60 min of ST treatment was more effective than a schedule of three sessions per week for 120 min of computer-based treatment. This discrepancy could potentially be attributed to the relative unfamiliarity of the included aphasic patients with computer programs (20). The study by Kesav et al. (20) did not provide detailed descriptions of the feedback mechanisms and patient compliance related to computer-based treatment. Personalized therapy and feedback are crucial factors for enhancing treatment efficacy (37). However, in our study, only 40% of the reports mentioned the feedback methods used in mobile application-based interventions, and these feedback mechanisms typically displayed errors made by patients during training. This type of feedback is relatively limited compared to the feedback provided by ST. Future research could explore whether more diverse feedback methods might have an impact on treatment outcomes.

Treatments that can be delivered at home can reduce the financial burden on patients and provide a higher intensity of treatment. Several studies have emphasized the need for intensive treatment programs for aphasia (38, 39). According to a network meta-analysis, the greatest gains in overall language proficiency were associated with >20 h of SLT (40). Breitenstein et al. observed that engaging in ≥10 h of intensive speech-language therapy per week for 3 weeks effectively improved communication skills in patients with chronic aphasia (41). Clearly, delivering therapy through mobile applications enables patients to achieve higher treatment intensity while participating in independent treatment. However, among the studies we included, only one managed to reach a treatment intensity of ≥10 h per week. Cherney et al. (26) founded that engaging in computer-based language imitation therapy six times per week, with each session lasting 90 min, effectively enhanced the overall language abilities of the patients. However, the training effects did not show significant differences when compared to the control group. This could potentially be attributed to the fact that the control group utilized computer games designed for memory and attention training. Training focused on cognitive functions might also lead to improvements in language abilities among individuals with aphasia (42). Therefore, it remains uncertain whether intensive, application-based SLT effectively enhances language function in individuals with aphasia. Additionally, application-based interventions offer a cost-effective supplemental approach that can address the limitations of high-intensity, in-clinic speech therapy for individuals who face challenges in accessing such treatment (43).

From the studies we included, the interventions can be divided into two main types: single-component interventions and multi-component interventions. Single-component interventions primarily focus on naming abilities. For instance, Palmer et al. (22) found that computer-based word retrieval training effectively improved naming abilities in individuals with aphasia, but did not significantly enhance their communicative skills. This might be attributed to the narrow focus of the training content. In contrast, Spaccavento et al. (23) employed a computer-based multi-component intervention, which resulted in significant improvements in the communicative abilities of aphasic patients post-treatment. However, as there have been no studies directly comparing the effectiveness of these two types of interventions, we cannot conclude whether there is a difference in the improvement effects between single-component and multi-component interventions. Additionally, the extent to which training effects can transfer to other functional aspects requires further investigation. Similar to findings in studies related to post-stroke motor function, where motor training improved motor skills but had limited effects on overall quality of life and daily functioning, it remains important to determine the potential for transfer effects in aphasia interventions (44, 45). In conclusion, further research is needed to delve into the differences in improvement effects between single-component and multi-component interventions, in order to better inform the application of mobile application-based speech-language therapy in clinical practice.

## 5 Limitation

The sample sizes of the included studies were generally small, ranging from 7 to 169 individuals, with ~66% of the studies having a sample size of <30 individuals. This small sample size may be related to the difficulty of recruiting people with aphasia. However, smaller sample sizes may affect the assessment of treatment effects. In addition, there was variability in the duration of post-stroke in aphasia patients in the included studies, with ~73% of the studies including patients with chronic aphasia, so there is uncertainty about the benefit of treatment for patients with different stages of aphasia. Another challenge was the wide variation in the intervention components used in the included studies, which made it difficult to compare and synthesize the findings. Finally, only eight studies explored the effect of mobile application-based intervention methods on the maintenance of efficacy, with follow-up times ranging from 2 weeks to 6 months, with an average of approximately 12 weeks. However, this follow-up period may be too short to effectively assess whether intervention approaches are able to maintain language functioning in people with aphasia over an extended period of time.

## 6 Recommendations for research

With regard to the many shortcomings and deficiencies of CSLT research, based on these studies, future researchers can develop a more rigorous and standardized procedure to validate the significance of CSLT beyond traditional face-to-face speech therapy. In this procedure, several aspects need consideration: the types and severity of aphasia; the optimal treatment stages of aphasia; maintenance therapy in chronic phases; the best monitoring of naming, perception, communication performance, social participation, and wellbeing; the definition of appropriate control groups; the efficacy and maintenance of intensified therapy; and the management of patient attention and feedback control interference. It is worth noting that the cost-effectiveness of CSLT is not publicly available and requires separate investigation.

## 7 Conclusion

The findings of this systematic review suggest that mobile application-based interventions for aphasia hold promise in improving overall language function. However, uncertainties remain regarding the improvement in functional communication abilities and whether gains in naming abilities can transfer to untrained objects. The efficacy of single-component interventions cannot be directly compared to multi-component interventions. Nonetheless, overall, mobile application-based interventions show positive prospects for enhancing speech-language function in aphasia.

## Data availability statement

The datasets presented in this study can be found in online repositories. The names of the repository/repositories and accession number (s) can be found in the article/Supplementary material.

## Author contributions

ZJ: Writing – original draft, Software, Methodology, Investigation, Formal Analysis, Data curation, Conceptualization. MH: Writing – review & editing, Supervision, Investigation. CZ: Writing – review & editing, Supervision, Software, Investigation. XC: Writing – review & editing, Visualization, Supervision, Resources, Project administration, Funding acquisition, Conceptualization.
