# Supplementary material for: The effect of mobile application-based technology on post-stroke aphasia: a systematic review
Source: Front Neurol. 2024 Jun 12;15:1405209. doi: 10.3389/fneur.2024.1405209 (PMC11199723; doi:10.3389/fneur.2024.1405209)
Supplement: Supplementary file 1 [file Data_Sheet_1.docx]

**The effect of mobile application-based technology on post-stroke aphasia：A systematic review**

**Additional file**

**Table S1. Searching Strategy**

Database: PubMed

| Set # |  | result |
| --- | --- | --- |
| 1 | stroke[MeSH Terms] or cerebrovascular disorders[MeSH Terms] or Ischemic Stroke[MeSH Terms] |  |
| 2 | stroke*[Title/Abstract] or post stroke*[Title/Abstract] or CVA*[Title/Abstract] or cerebrovascular$[Title/Abstract] or cerebral vascular[Title/Abstract] or cerebral[Title/Abstract] or cerebellar[Title/Abstract] or brain$[Title/Abstract] or vertebrobasilar[Title/Abstract] or apoplexy[Title/Abstract] or intracerebral[Title/Abstract] or intracranial[Title/Abstract] or brain$[Title/Abstract] or haemorrhage[Title/Abstract] |  |
| 3 | #1 or #2 |  |
| 4 | Aphasia[MeSH Terms] OR language disorders[MeSH Terms] OR speech disorders[MeSH Terms] |  |
| 5 | Aphasia[Title/Abstract] OR anomic[Title/Abstract] OR anomia[Title/Abstract] OR language disorders[Title/Abstract] OR speech disorders[Title/Abstract] OR alogia[Title/Abstract] |  |
| 6 | #4 or #5 |  |
| 7 | Computer[Title/Abstract] OR computer training[Title/Abstract] OR computer therapy[Title/Abstract] OR computer support[Title/Abstract] OR computer treatment[Title/Abstract] OR computer rehabilitation[Title/Abstract] OR computerized[Title/Abstract] OR computerised[Title/Abstract] OR computer-based[Title/Abstract] OR computer-mediated[Title/Abstract] OR ipad[Title/Abstract] OR ipad-based[Title/Abstract] OR ipad-mediated[Title/Abstract] OR tablet[Title/Abstract] OR tablet-based[Title/Abstract] OR Tablet-mediated[Title/Abstract] OR mobile-based[Title/Abstract] OR app[Title/Abstract] OR app support[Title/Abstract] OR app training[Title/Abstract] |  |
| 8 | #3 and #6 and #7 | 477 |

Database: Web of Science

| Set # |  | result |
| --- | --- | --- |
| 1 | TS=(stroke* OR cerebrovascular disorders OR Ischemic Stroke OR post stroke* OR cva* OR cerebrovascular$ OR cerebral vascular OR cerebral OR cerebellar OR brain$ OR vertebrobasilar OR apoplexy OR cerebral OR intracerebral OR intracranial OR haemorrhage) |  |
| 2 | TS=(Computer OR computer training OR computer therapy OR computer support OR computer treatment OR computer rehabilitation OR computerized OR computerised OR computer-based OR computer-mediated OR ipad OR ipad-based OR ipad-mediated OR tablet OR tablet-based OR Tablet-mediated OR mobile-based OR app OR app support OR app training) |  |
| 3 | TS=(Aphasia OR anomic OR anomia OR language disorders OR speech disorders OR alogia) |  |
| 4 | #1 and #2 and #3 | 708 |

Database: EMBASE

| Set # |  | result |
| --- | --- | --- |
| 1 | 'cerebrovascular accident'/exp OR 'cerebrovascular accident' OR 'stroke*':ab,ti OR 'post stroke*':ab,ti OR 'cerebrovascular disorders':ab,ti OR 'ischemic stroke':ab,ti OR 'cva*':ab,ti OR 'cerebrovascular$':ab,ti OR 'vascular':ab,ti OR 'cerebral':ab,ti OR 'cerebellar':ab,ti OR 'vertebrobasilar':ab,ti OR 'apoplexy':ab,ti OR 'intracerebral':ab,ti OR 'intracranial':ab,ti OR 'brain$':ab,ti OR 'haemorrhage':ab,ti |  |
| 2 | 'aphasia'/exp OR 'aphasia' OR 'aphasia':ab,ti OR 'anomic':ab,ti OR 'anomia':ab,ti OR 'language disorders':ab,ti OR 'speech disorders':ab,ti OR 'alogia':ab,ti |  |
| 3 | ('computer':ab,ti OR 'computer training':ab,ti OR 'computer therapy':ab,ti OR 'computer support':ab,ti OR 'computer treatment':ab,ti OR 'computer rehabilitation':ab,ti OR 'computerized':ab,ti) AND 'computerised':ab,ti OR 'computer-based':ab,ti OR 'computer-mediated':ab,ti OR 'ipad':ab,ti OR 'ipad-based':ab,ti OR 'ipad-mediated':ab,ti OR 'tablet':ab,ti OR 'tablet-based':ab,ti OR 'tablet-mediated':ab,ti OR 'mobile-based':ab,ti OR 'app':ab,ti OR 'app support':ab,ti OR 'app training':ab,ti |  |
| 4 | #1 and #2 and #3 | 187 |

Database: CENTRAL

| Set # |  | result |
| --- | --- | --- |
| 1 | TS=(stroke* OR cerebrovascular disorders OR Ischemic Stroke OR post stroke* OR cva* OR cerebrovascular$ OR cerebral vascular OR cerebral OR cerebellar OR brain$ OR vertebrobasilar OR apoplexy OR cerebral OR intracerebral OR intracranial OR haemorrhage) |  |
| 2 | TS=(Computer OR computer training OR computer therapy OR computer support OR computer treatment OR computer rehabilitation OR computerized OR computerised OR computer-based OR computer-mediated OR ipad OR ipad-based OR ipad-mediated OR tablet OR tablet-based OR Tablet-mediated OR mobile-based OR app OR app support OR app training) |  |
| 3 | TS=(Aphasia OR anomic OR anomia OR language disorders OR speech disorders OR alogia) |  |
| 4 | #1 and #2 and #3 | 11 |

Database: Scopus

| Set # |  | result |
| --- | --- | --- |
| 1 | TITLE-ABS-KEY ( stroke* OR cerebrovascular disorders OR Ischemic Stroke OR post stroke* OR cva* OR cerebrovascular$ OR cerebral vascular OR cerebral OR cerebellar OR brain$ OR vertebrobasilar OR apoplexy OR cerebral OR intracerebral OR intracranial OR haemorrhage) |  |
| 2 | TITLE-ABS-KEY (Computer OR computer training OR computer therapy OR computer support OR computer treatment OR computer rehabilitation OR computerized OR computerised OR computer-based OR computer-mediated OR ipad OR ipad-based OR ipad-mediated OR tablet OR tablet-based OR Tablet-mediated OR mobile-based OR app OR app support OR app training) |  |
| 3 | TITLE-ABS-KEY (Aphasia OR anomic OR anomia OR language disorders OR speech disorders OR alogia) |  |
| 4 | #1 and #2 and #3 | 8 |

**Content 1:** **Detials of Cochrane Risk of Bias Tool**

**Selection bias: random sequence generation**

⦁ Low risk of bias：Researchers have described random methods such as: random number tables, computer generated random numbers, coin tossing method, shuffling of cards or envelopes, rolling of dice, drawing of lots, minimization, etc. in the process of sequence generation;

⦁ Uncertain risk of bias: Sequence generation information is not available, making it difficult to determine whether it is "low bias" or "high bias";

⦁ High risk of bias：The researcher describes a non-randomized approach in the sequence generation process.

**Selection bias: allocation concealment**

⦁ Low risk of bias：Subjects or investigators recruiting subjects cannot predict allocation because of the use of the following reasons or equivalent methods to hide the randomized allocation scheme: central allocation (including telephone, website, and pharmacy-controlled randomization); identically shaped and ordered containers of medication; ordered opaque sealed envelopes;

⦁ Uncertain risk of bias: Insufficient information to judge "low bias" or "high bias". Often hidden methods are not described or are not sufficiently described to give a clear judgment;

⦁ High risk of bias：Subjects or researchers recruiting subjects may be able to anticipate allocation and cause selective bias.

**Performance bias: blinding participants and personnel**

⦁ Low risk of bias：Presence of any of the following: no blinding or inadequate blinding, but the systematic evaluator judges that the outcome will not be affected by the absence of blinding; blinding of subjects and principal investigators, and the blinding will not be compromised;

⦁ Uncertain risk of bias: The presence of any of the following: insufficient information for a "yes" or "no" judgment; the outcome indicator was not reported in the study;

⦁ High risk of bias：Presence of any of the following: blinding is not used or is imperfect, and outcome judgments or measurements are affected; blinding is applied to the outcome measures, but this blinding may be compromised.

**Detection bias: blinding of outcome assessors**

⦁ Low risk of bias：Presence of any of the following: blinding is not applied, but the system evaluator judges that the outcome measure will not be affected by the absence of blinding; blinding is applied to the outcome measurer and the blinding will not be disrupted;

⦁ Uncertain risk of bias: Presence of any of the following: insufficient information for a "yes" or "no" judgment; the outcome indicator was not reported in the study;

⦁ High risk of bias：Presence of any of the following: blinding is not used or is imperfect, and outcome judgments or measurements are affected; blinding is applied to the outcome measures, but this blinding may be compromised.

**Attrition bias: incomplete outcome data**

⦁ Low risk of bias：: Missing data are unlikely to bias treatment effects away from plausible values. Missing data were dealt with using methods such as multiple interpolation;

⦁ Uncertain risk of bias: Incomplete information, making it difficult to judge the completeness of the data (e.g., number of people missing or reasons not reported); the study does not address the issue of completeness;

⦁ High risk of bias：Results may be biased due to missing data.

**Reporting bias**

⦁ Low risk of bias：There was a study protocol and the systematic evaluation reported all predefined outcome indicators (primary and secondary endpoints) as intended; there was no study protocol, but all desired endpoints (which included predefined endpoints) were reported in the published study, including those that were predefined;

⦁ Uncertain risk of bias: If there is doubt as to whether the reported results include all measurements;

⦁ High risk of bias：Not all prespecified key outcome indicators were reported; one or more of the key outcome indicators were reported using prespecified measurements, data analyses, etc.

**Other potential sources of bias**

⦁ Low risk of bias：The trial does not appear to be otherwise biased;

⦁ Uncertain risk of bias: There was insufficient information to determine whether the trial was free of other biases;

⦁ High risk of bias：There is at least one significant risk of bias. E.g., the study had potential bias related to the particular study design; the statement was falsified; some other issue.

**Table S2 For PEDro-based qualitative evaluation of randomized controlled trials**

| study | Eligibility criteria and source | Random allocation | Concealed allocation | Baseline comparability | Blinding of participants | Blinding of therapists | Blinding of assessors | Adequate follow-up (. 85%) | Intention-to-treat analysis | Between-group statistical comparisons | Reporting of point measures and measures of variability | overall |
| --- | --- | --- | --- | --- | --- | --- | --- | --- | --- | --- | --- | --- |
| Braley，2021 | 1 | 1 | 0 | 1 | 0 | 0 | 1 | 1 | 1 | 1 | 1 | 7 |
| Cherney，2010 | 1 | 1 | 0 | 1 | 0 | 0 | 1 | 1 | 1 | 1 | 0 | 6 |
| Cherney，2021 | 1 | 1 | 0 | 1 | 0 | 0 | 0 | 1 | 1 | 1 | 0 | 6 |
| Elhakeem，2021 | 1 | 1 | 1 | 1 | 0 | 0 | 1 | 1 | 1 | 1 | 1 | 8 |
| Kesav，2017 | 1 | 1 | 0 | 1 | 0 | 0 | 1 | 1 | 1 | 1 | 0 | 6 |
| Palmer，2019 | 1 | 1 | 1 | 1 | 0 | 0 | 1 | 1 | 1 | 1 | 1 | 8 |
| Palmer，2012 | 1 | 1 | 0 | 1 | 0 | 0 | 1 | 1 | 1 | 0 | 0 | 5 |
| Spaccavento，2021 | 1 | 1 | 0 | 1 | 0 | 0 | 1 | 1 | 1 | 1 | 0 | 6 |
| Doesborgh，2004 | 1 | 1 | 1 | 1 | 0 | 0 | 0 | 1 | 1 | 1 | 0 | 6 |
| Katz，1997 | 1 | 0 | 0 | 1 | 0 | 0 | 0 | 1 | 1 | 1 | 0 | 4 |

**Table S3:TREND (Transparent Reporting of Evaluations with Nonrandomized Designs) Checklist**

| **Paper**  **Section/**  **Topic** | **Item**  **No.** | **Descriptor** | **Archibald，2009** | | **Choi,2016** | | **Zettin，2019** | | **Kurland，2018** | | **Stark，2016** | |
| --- | --- | --- | --- | --- | --- | --- | --- | --- | --- | --- | --- | --- |
|  |  |  | 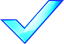 | Pg # | 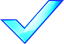 | Pg # | 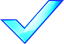 | Pg # | 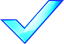 | Pg # | 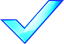 | Pg # |
| **TITLE AND ABSTRACT** | | | | | | | | | | | | |
| **Title and Abstract** | **1** | ⦁Information on how unit were allocated to interventions | Yes | 1 | Yes | 1 | Yes | 1 | Yes | 1 | Yes | 1 |
|  |  | ⦁Structured abstract recommended | Yes | 1 | Yes | 1 | Yes | 1 | Yes | 1 | Yes | 1 |
|  |  | ⦁Information on target population or study sample | Yes | 1 | Yes | 1 | Yes | 1 | Yes | 1 | Yes | 1 |
| **INTRODUCTION** | | | | | | | | | | | | |
| **Background** | **2** | ⦁Scientific background and explanation of rationale | Yes | 2 | Yes | 1 | Yes | 4 | Yes | 2 | Yes | 3 |
|  |  | ⦁Theories used in designing behavioral interventions | Yes | 2 | Yes | 1 | Yes | 4 | Yes | 2 | Yes | 3 |
| **METHODS** | | | | | | | | | | | | |
| **Participants** | **3** | ⦁Eligibility criteria for participants | Yes | 3 | Yes | 2 | Yes | 8 | No |  | Yes | 4 |
|  |  | ⦁Method of recruitment | No |  | No |  | No |  | No |  | No |  |
|  |  | ⦁Recruitment setting | Yes | 3 | Yes | 2 | No |  | No |  | Yes | 4 |
|  |  | ⦁Settings and locations where the data were collected. | No |  | Yes | 2 | No |  | No |  | Yes | 4 |
| **Interventions** | **4** | ⦁Details of the interventions intended for each study condition and how and when they were actually administered, specifically including: |  |  |  |  |  |  |  |  |  |  |
|  |  | o Content: what was given? | Yes | 4 | Yes | 2 | Yes | 6 | Yes | 4 | Yes | 5 |
|  |  | o Delivery method: how was the content given? | Yes | 4 | Yes | 2 | Yes | 6 | Yes | 4 | Yes | 5 |
|  |  | o Unit of delivery: how were the subjects grouped during delivery? | Yes | 4 | Yes | 2 | Yes | 6 | Yes | 5 | Yes | 5 |
|  |  | o Deliverer: who delivered the intervention? | Yes | 4 | Yes | 2 | Yes | 6 | Yes | 5 | Yes | 5 |
|  |  | o Setting: where was the intervention delivered? | Yes | 4 | No |  | Yes | 12 | Yes | 6 | Yes | 5 |
|  |  | o Exposure quantity and duration | Yes | 4 | Yes | 2 | Yes | 11 | Yes | 6 | Yes | 5 |
|  |  | o Time span: | Yes | 4 | Yes | 2 | Yes | 11 | Yes | 1 | Yes | 5 |
|  |  | o Activities to increase compliance or adherence (e.g., incentives) | No |  | No |  | No |  | No |  | No |  |
| **Objectives** | **5** | ⦁Specific objectives and hypotheses | Yes | 3 | Yes | 1 | Yes | 5 | Yes | 3 | No |  |
| **Outcomes** | **6** | ⦁Clearly defined primary and secondary outcome measures | No |  | No |  | Yes | 7 | Yes | 6 | No |  |
|  |  | ⦁Methods used to collect data and any methods used to enhance the quality of measurements | No |  | No |  | No |  | No |  | No |  |
|  |  | ⦁Information on validated instruments such as psychometric and biometric properties | Yes | 5 | No |  | No |  | No |  | No |  |
| **Sample Size** | **7** | ⦁How sample size was determined and, when applicable, explanation of any interim analyses and stopping rules | No |  | No |  | No |  | No |  | No |  |
| **Assignment Method** | **8** | ⦁Unit of assignment | Yes | 3 | Yes | 1 | No |  | Yes | 3 | Yes | 6 |
|  |  | ⦁Method used to assign units to study conditions | No |  | No |  | No |  | No |  | No |  |
|  |  | ⦁Inclusion of aspects employed to help miniminze potential bias induced due to non-randomization | No |  | No |  | No |  | No |  | No |  |
| **Blinding** | **9** | ⦁Whether or not participants, those administering the interventions, and (masking) those assessing the outcomes were blinded to study condition assignment; if so, statement regarding how the blinding was accomplished and how it was assessed. | No |  | No |  | No |  | No |  | No |  |
| **Unit of Analysis** | **10** | ⦁Description of the smallest unit that is being analyzed to assess intervention effects | Yes | 3 | Yes | 2 | Yes | 8 | Yes | 3 | Yes | 4 |
|  |  | ⦁If the unit of analysis differs from the unit of assignment, the analytical method used to account for this | No |  | No |  | No |  | No |  | No |  |
| **Statistical** | **11** | ⦁Statistical methods used to compare study groups for primary methods. Methods outcome(s), including complex methods of correlated data | Yes | 6 | Yes | 2 | Yes | 14 | Yes | 7 | No |  |
|  |  | ⦁Statistical methods used for additional analyses, such as a subgroup analyses and adjusted analvsis | No |  | No |  | No |  | No |  | No |  |
|  |  | ⦁Methods for imputing missing data, if used | No |  | No |  | No |  | No |  | No |  |
|  |  | ⦁Statistical software or programs used | Yes | 6 | Yes | 4 | No |  | No |  | No |  |
|  | | | | | | | | | | | | |
| **Participant flow** | **12** | ⦁Flow of participants through each stage of the study: enrollment, assignment, allocation and intervention exposure, follow-up, analysis |  |  |  |  |  |  |  |  |  |  |
|  |  | o Enrollment: the numbers of participants screened for eligibility, found to be eligible or not eligible, declined to be enrolled, and enrolled in the study | No |  | No |  | No |  | No |  | Yes | 4 |
|  |  | o Assignment: the numbers of participants assigned to a study condition | Yes | 4 | Yes | 4 | Yes | 8 | Yes | 3 | Yes | 4 |
|  |  | o Allocation and intervention exposure: the number of participants assigned to each study condition and the number of participants who received each intervention | Yes | 4 | Yes | 4 | Yes | 8 | Yes | 3 | Yes | 4 |
|  |  | o Follow-up: the number of participants who completed the follow-up or did not complete the follow-up (i.e., lost to follow-up), by study condition | No |  | Yes | 4 | No |  | No |  | No |  |
|  |  | o Analysis: the number of participants included in or excluded from the main analysis, by study condition | Yes | 4 | No |  | No |  | No |  | No |  |
|  |  | ⦁Description of protocol deviations from study as planned, along with reasons | No |  | No |  | No |  | No |  | No |  |
| **Recruitment** | **13** | ⦁Dates defining the periods of recruitment and follow-up | No |  | Yes | 2 | No |  | No |  | Yes | 6 |
| **Baseline data** | **14** | ⦁Baseline demographic and clinical characteristics of participants in each study condition | Yes | 7 | Yes | 4 | Yes | 9 | Yes | 4 | Yes | 6 |
|  |  | ⦁Baseline characteristics for each study condition relevant to specific disease prevention research | No |  | No |  | No |  | No |  | No |  |
|  |  | ⦁Baseline comparisons of those lost to follow-up and those retained, overall and by study condition | No |  | No |  | No |  | No |  | No |  |
|  |  | ⦁Comparison between study population at baseline and target population of interest | No |  | No |  | No |  | No |  | Yes | 8 |
| **Baseline equivalence** | **15** | ⦁Data on study group equivalence at baseline and statistical methods used to control for baseline differences |  |  |  |  |  |  |  |  | No |  |
| **Numbers analyzed** | **16** | ⦁Number of participants (denominator) included in each analysis for each study condition, particularly when the denominators change for different outcomes; statement of the results in absolute numbers when feasible | No |  | Yes | 4 | Yes | 8 | Yes | 3 | Yes | 8 |
|  |  | ⦁Indication of whether the analysis strategy was “intention to treat”or, if not, description of how noncompliers were treated in the analyses | No |  | No |  | No |  | No |  | No |  |
| **Outcomes and estimation** | **17** | ⦁For each primary and secondary outcome, a summary of results for each study condition, and the estimated effect size and a confidence interval to indicate the precision | No |  | No |  | No |  | No |  | No |  |
|  |  | ⦁Inclusion of null and negative findings | Yes | 9 | Yes | 5 | Yes | 14 | Yes | 8 | Yes | 8 |
|  |  | ⦁Inclusion of results from testing prespecified causal pathways through which the intervention was intended to operate, if any | No |  | No |  | No |  | No |  | No |  |
| **Ancillary analyses** | **18** | ⦁Summary of other analyses performed, including subgroup or restricted analyses, indicating which are prespecified or exploratory | No |  | No |  | No |  | No |  | No |  |
| **Adverse events** | **19** | ⦁Summary of all important adverse events or unintended effects in each study condition | No |  | No |  | No |  | No |  | No |  |
|  | | | | | | | | | | | | |
| **Interpretation** | **20** | ⦁Interpretation of the results, taking into account study hypotheses, sources of potential bias, imprecision of measures, multiplicative analyses, and other limitations or weaknesses of the study | Yes | 12 | Yes | 6 | Yes | 23 | Yes | 14 | No |  |
|  |  | ⦁Discussion of results taking into account the mechanism by which the intervention was intended to work (causal pathways) or alternative mechanisms or explanations | Yes | 11 | No |  | No |  | No |  | No |  |
|  |  | ⦁Discussion of the success of and barriers to implementing the intervention, fidelity of implementation | Yes | 11 | Yes | 6 | Yes | 23 | Yes | 14 | Yes | 12 |
|  |  | ⦁Discussion of research, programmatic, or policy implications | Yes | 11 | Yes | 6 | No |  | Yes | 14 | Yes | 12 |
| **Generalizability** | **21** | ⦁Generalizability (external validity) of the trial findings, taking into account the study population, the characteristics of the intervention,length of follow-up, incentives, compliance rates, specific sites/settings involved in the study, and other contextual issues | No |  | No |  | No |  | No |  | No |  |
| **Overall evidence** | **22** | ⦁General interpretation of the results in the context of current evidence and current theory | Yes | 12 | Yes | 6 | No |  | Yes | 14 | No |  |

**Table S4** **Detailed results of the included literature**

| First author,year | Outcome measures | Result | conclusion |
| --- | --- | --- | --- |
| Archibald，2009 | WAB | WAB-AQ：  Pre to post-treatment change 6.34±10.11, Z=－1.54, p=0.123;  auditory comprehension of WAB：  Pre to post-treatment change 0.78±0.64, Z=－2.18, p=0.03; | study participants improved significantly and reliably on standardized measures of  auditory comprehension. Other outcomes were not significantly different after treatment. |
|  | CETI | Pre to post-treatment change 9±9.72, Z=－1.75，p=0.08； |  |
|  | ASHA-FACS | QDC of ASHAFACS: Z=－2.38, p=0.017;  CIM of ASHAFACS: Z=0.73, p=0.46. |  |
| Braley，2021 | WAB-R | WAB-R-AQ：  IG 68.37±26.24, CG 66.40±20.22, p<0.01；  WAB-R-LQ ：  IG 69.15±25.33, CG 66.57±21.51, p<0.01；  WAB-R-CQ ：  IG 72.38±23.27, CG 69.59±19.00, p<0.05. | Compared with the CG, IG showed a significant improvement in WAB-R-AQ, WAB-R-LQ and WAB-R-CQ. But there was no significant improvement in SAQOL-39. |
|  | SAQOL-39 | IG 3.77±0.56, CG 3.66±0.70, p=0.35. |  |
| Cherney，2010 | WAB | WAB-AQ(change between baseline and posttreatment):  IG 3.29 ±6.16, CG -0.4±3.44, d=0.74, p>0.05.  WAB reading(change between baseline and posttreatment)：  IG −3.55±13.16, CG 1.36±12.80, d=-0.38, p>0.05.  WAB writing(change between baseline and posttreatment):  IG 0.35±7.72, CG 0±5.80, d=0.05, p>0.05. | Treatment with IG improved both WAB and speech content performance, but there was no significant difference in outcomes between IG and CG. |
|  | Discourse words/min | IG 7.48±6.51, CG 0.83±9.56, d=0.81, p>0.05. |  |
|  | Discourse CIUs/min | IG 4.34±5.36, CG 2.04±4.43, d=0.47, p>0.05. |  |
| Choi,2016 | K-WAB-AQ | **WAB-AQ:**  Pre 49.63±28.20, post 57.00±23.80, p = 0.027  1-month follow: 58.63±24.45, p=0.206.  **WAB fluency:**  Pre 44.63±24.38, post 52.25±21.67, p=0.039  1-month follow: 54.88±24.50, p=0.141.  **WAB Auditory Comprehension**:  Pre 53.00±33.13, post 64.50±26.74, p=0.028.  1-month follow: 65.88±25.83, p=0.553.  **WAB repetition**:  Pre 52.75±32.76, post 55.13±29.04, p=0.398.  1-month follow: 56.38±29.79, p=0.398.  **WAB naming**:  Pre 53.8±26.40, post 61.50±21.17, p=0.075.  1-month follow: 64.75±22.45, p=0.207.  **WAB reading:**  Pre 61.00±29.68, post 64.13±25.76, p=0.225  1-month follow: 73.50±21.77, p=0.018.  **WAB writing**:  Pre 59.38±23.54, post 67.13±23.51, p=0.116.  1-month follow: 68.75±23.00, p=0.202. | WAB scores improved significantly before and after treatment, and efficacy was maintained during follow-up. |
| Elhakeem，2021 | BADE | Insignificant for most of results (p>0.05) | There was no significant difference in outcomes between IG and CG. |
| Kesav，2017 | WAB(rate of rise of AQ) | Base：IG 45.1±28.4, CG 32.4±25.8.  4 weeks post treatment: IG 63.5±33.8, CG 65.1±27.6.  8 weeks follow up: IG 67.6±32.7, CG 73.7±26.9.  Base VS 4 weeks follow up: p=0.053 | IG's improvement in WAB was not significantly higher than CG's. |
| Palmer，2019 | picture  naming test of 100 personally relevant words | mean difference in change (95% CI)：14.4 (10.8 to 18.1)，p<0.0001  6month follow up: Improvement was maintained | IG's naming ability is significantly improved compared to CG, but IG's communication ability is not significantly different from CG's. |
|  | functional communication ability | mean difference in change (95% CI)：–0·01 (–0·20 to 0·18), p=0.915 |  |
|  | COAST | mean difference in change (95% CI)：3·8 (–0·0 to 7·5), p=0.051 |  |
| Palmer，2012 | the change in word retrieval ability | mean difference in change(95% CI)：19.8%（4.4%-35.2%），P=0.014  3-month follow up: No significant different from baseline | IG's naming ability is significantly improved compared to CG. After 3 months of follow-up, the curative effect was not maintained. |
| Spaccavento，2021 | AAT | **AAT -Spontaneous Language**:  IG 14.85 ± 10.8, CG 18.11 ± 7.32. F=0.06, P=0.81.  **AAT-Token Test**:  IG 26 ± 19.49, CG 24.22 ± 8.81. F=0.03, P=0.87.  **AAT- Repetition**：  IG 87.15 ± 67.69, CG 104 ± 35.99. F=0.001, P=0.97.  **AAT- Writing**:  IG 46.31 ± 39.71, CG 40.78 ± 32.67. F=0.33, P=0.57.  **AAT-Naming**:  IG 59.46 ± 51.3, CG 74 ± 39.91. F=0.17, P=0.68.  **AAT-Comprehension**:  IG 82.38 ± 36.5, CG 95.67 ± 12.9. F=0.85, P=0.37. | IG showed significant improvement in speech function before and after treatment, but there was no significant difference in outcome compared with CG. |
|  | FOQ-A | IG 102.16 ± 51.22, CG 115.25 ± 38.51. F=0.17, P=0.69. |  |
|  | QLQA | IG 59.66 ± 40.49, CG 72 ± 24.45. F=0.65, P=0.43. |  |
| Doesborgh，2004 | BNT | IG 75.6±38.7, CG 75.7±36.7. p=1.0 | IG showed significant improvement in BNT before and after treatment, but there was no significant difference in BNT and ANELT-A compared with CG. |
|  | ANELT-A | IG 34.3±8.4, CG 25.5±10.3. p=0.07 |  |
| Zettin，2019 | WAB | WAB-AQ:  Pre: 43±16.6, post 54.2±13.3. p=0.001  WAB- Spontaneous Language:  Pre 6.4±3.7, post 8.6±2.5. p=0.023.  WAB- Comprehension:  Pre 7.1±1.6, post 8.1±1.2. p=0.04  WAB- repetition:  Pre 4.4±1.4, post 5.5±1.3. p=0.016  WAB-naming:  Pre 3.8±2.7, post 4.9±2.6. p=0.013. | A significant improvement in WAB, BNT and picture description task before and after treatment. |
|  | BNT | Pre 0.2±0.2, post 0.4±0.2. p=0.001. |  |
|  | picture description task | pre 0.2±0.2, post 0.4±0.2. p=0.007. |  |
| Kurland，2018 | percent accuracy on naming | For treated pictures，11% gain for those with severe, 17% gain for those with mod/sev, and 15% gain for those with mild/mod. Improvements can be sustained over time.  For untreated pictures, naming gains also evident but not statistically significant for those with severe aphasia over time. | After treatment, the improvement was greater in patients with mild and moderate aphasia and less in patients with severe aphasia. |
| Stark，2016 | CAT expressive | Post- speech therapy, IG showed a very large effect size for therapy compared to baseline (d = 2.730).  Follow up: maintained. | IG showed significant improvement over CAT and content unit production and rate of speech. |
|  | content unit production and rate of speech on the CTPD | Post-speech therapy(d = 0.448). Post game therapy (d = 0.365).  Follow up: maintained. |  |
| Katz，1997 | PICA | PICA overall:  IG changed 9.1, CG changed 4.4, p<0.003.  PICA verbal:  IG changed 7.9, CG changed 1.3. p<0.001. | IG showed significant improvement over WAB-AQ, WAB repetition, PICA overall and PICA verbal compared to CG. |
|  | WAB-AQ | WAB-AQ:  IG changed 4.7, CG changed 1.5. p<0.008.  WAB repetition:  IG changed 0.6, CG changed 0.1. p<0.021. |  |
| Cherney，2021 | WAB-LQ | Pre to post-treatment: 0.99±1.41, p=0.448  Pre to six-week follow-up:2.70 ±1.01, p=0.013.  IG compared to CG: no significant. | IG showed significant improvement in WAB-LQ before and after treatment, but there was no significant difference compared with CG. |

IG: Intervention group, CG Control group, m=months, d=days, y=years, WAB= Western Aphasia Battery, R=Revised, AQ= Aphasia Quotient, CQ= Cortical Quotients, LQ=Language Quotients, SAQOL= Stroke and Aphasia Quality of Life Scale, CIU= correct information units, BADE= Boston Diagnostic Aphasia Examination, COAST= Communication Outcomes After Stroke questionnaire, AAT= Aachener Aphasia Test, FOQ-A= Italian Version of Functional Outcome Questionnaire for Aphasia, QLQA= Quality of Life Questionnaire for Aphasics, BNT= Boston Naming Test, ANELT-A=Amsterdam Nijmegen Everyday Language Test, PICA= The Porch Index of Communicative Ability, CETI= Communicative Effectiveness Index, ASHA-FACS= Functional Assessment of Communication Skills for Adults, CAT= Comprehensive Aphasia Test, CTPD= Cookie Theft Picture Description.

**Table S5 Detailed description of the intervention methods included in the study**

| First author,year | Intervention description | Description of the difficulty level | Description of feedback |
| --- | --- | --- | --- |
| Archibald，2009 | AphasiaMate app(computer-based)：training in 8 aspects of auditory processing, visual matching, reading comprehension, spelling, semantic processing, sentence processing, time and money processing. | Not clear | Not clear |
| Braley，2021 | Constant therapy app(tablet-based): over 100,000 stimuli across 9 different cognitive, speech and language domains. | This program has over 350 difficulty levels. | Not clear |
| Cherney，2010 | Computer stimulation: pre-recorded sentences of varying difficulty (length) by SLP, in which the patient reads the sentences and learns the words in the sentences with the help of the computer.  Procedure:  1. A speech language pathologist (SLP) on a computer reads aloud to the patient, who silently follows. The length of the material can range from 3 to 100 words.  2. The SLP on the computer reads aloud to the patient again.  3. The SLP reads the paragraph aloud with the patient, while continuing to point out each word as the patient reads. The patient will point to each word.  4. For each line or sentence of a paragraph, the SLP will point out a word and ask the patient to read it aloud.  5. The patient reads the entire sentence aloud again, consistent with SLP. | Not clear | Not clear |
| Choi,2016 | iAphasia(iPad-based): Six treatment programs are included: auditory comprehension, reading comprehension, repetition, naming, writing, and verbal fluency. | Each domain  had six levels of difficulty | This program used a grading system that provides automatic feedback to the patients and tracks improvement by correct answer rate graphs in the six domains. |
| Elhakeem，2021 | Rawag app(computer-based): It covers listening comprehension, naming training, sentence structure training, oral expression training, writing, arithmetic training and other training forms. | Researchers determined 90% response rate to move from one step to another in therapy | The program provides visual and or auditory feedback after getting the answers. In visual feedback, the correct answer appears green and the wrong answer appears red. |
| Kesav，2017 | MOZHI app(computer-based)：The hexarchial language hierarchy modules utilised in this software included a) Auditory verbal comprehension (body parts, common objects, food items, Y es/No questions with sequential commands); b) Expression of language assessment (response to general questions); c) Naming (confrontational naming, categorical naming); d) Writing (copying, dictation); e) Reading (matching tasks, reading commands, sentence completion, story reading, functional reading) and f) Calculation (addition, subtraction, multiplication, et al). | Not clear | Not clear |
| Palmer，2019 | word-finding exercises which were tailored to the needs of the individual patient by a qualified speech and language therapist  experienced in working with patients with stroke in  routine clinical practice. Each patient chose 100 words that were relevant to them, before they were randomly assigned to one of the intervention groups, which were then used for computerised word finding practice. | Not clear | Provide feedback  about whether the words used are correct. |
| Palmer，2012 | StepbyStep app(computer-based)：The computer program contains a library of 13,000 language exercises. Photo images can be added in order to practice words related to individuals, such as the names of people and pets. Each exercise follows steps from listening to the target word, producing a word with visual, semantic, phonemic, or written letter/word cues, to saying the word in a sentence. | Not clear | Not clear |
| Spaccavento，2021 | A app: program included exercises of verbal and written naming of nouns, verbs, single word and sentence level spoken word, naming of visual target, word retrieval, sentence building, and language comprehension. | The participant needed to achieve an accuracy rate of  80%. | At the end of each rehabilitation session, a table with the number of correct and incorrect responses was shown on the computer screen. |
| Zettin，2019 | IMITAF, Each participant sat in front of a computer screen and was asked to carefully observe six actors while pronouncing aloud a word or a sentence. Every word/sentence was pronounced by all the actors, so that it was repeated six times consecutively. After having observed the actors, the participant was asked to repeat what he/she listened to. | Difficulty is measured by the length of words and sentences. The passage from one to the following level of the program was decided by the clinical neuropsychologist. | The computer program did not give feedback on whether the performance was good or bad, and the caregivers' feedback was not described. |
| Kurland，2018 | iBooks(Tablet-Based): Individualized interactive books were created for each participant using iBooks Author software. Two books of 20 chapters were created for each PWA, one containing OBJECTION and one containing ACTION. Each chapter contained a title page with the name of the OBJECTION and four interactive pages targeting various levels of semantic, phonologic, and orthographic cueing. | Not clear | Not clear |
| Stark，2016 | Tactus Therapy(iPad-based): Reading, Naming, Comprehension and Writing. Within these four categories were several tasks that patients chose to complete. The type of training provided was both phonological and semantic in nature. For example, in the Naming: Describe exercise, hierarchical cues provided both semantic and phonological cueing for successful picture naming. There were 700+ core nouns, verbs and adjectives throughout the app. | The app adapted difficulty  as the user attained more correct answers. | The app provided feedback to the patient (correct, incorrect). |
| Katz，1997 | Computer treatment: The computer reading treatment software consisted of 10 matching activities (e.g., matching letters, matching words) and 22 reading comprehension activities. The matching activities (i.e., the first 80 tasks) were perceptual visual-matching activities designed to familiarize subjects with the operation of the program, format of the tasks, and displayed characters (letters, numbers, and symbols). Stimuli for the remaining reading comprehension activities (i.e., 152 tasks) consisted of letters, numbers, words, phrases, and sentences. | Each of the 10 matching activities had eight levels of difficulty. The entire program comprised 232 different tasks. | Not clear |
| Cherney，2021 | Computer treatment: The participant was presented with three to five word (level 1) or eight to ten word (level 2) sentences. Each sentence was chosen by the software program at random from a group of 150 sentences. The participant was instructed to look, listen, and point to words spoken by the virtual therapist, read highlighted words aloud, and then read the sentence aloud, both chorally with the virtual therapist and independently. | Difficulty is measured by the length of sentences. | Not clear |
| Doesborgh，2004 | Multicue: The 80 images were randomly presented and named according to the computer's prompts. | Not clear | Not clear |
